# CMV prophylaxis with letermovir significantly improves graft and relapse free survival following allogeneic stem cell transplantation

**DOI:** 10.1038/s41409-023-02124-y

**Published:** 2023-10-19

**Authors:** Michele Malagola, Vera Radici, Mirko Farina, Simone Pellizzeri, Filippo Spoldi, Enrico Morello, Nicola Polverelli, Eugenia Accorsi Buttini, Simona Bernardi, Federica Re, Alessandro Leoni, Liana Signorini, Arnaldo Caruso, Domenico Russo

**Affiliations:** 1 Blood Diseases and Cell Therapies Unit, Bone Marrow Transplant Unit, “ASST-Spedali Civili” Hospital of Brescia; Department of Clinical and Experimental Sciences, University of Brescia, Brescia, Italy; 2 Centro di Ricerca Emato-oncologico AIL (CREA), “ASST-Spedali Civili” Hospital, Brescia, Italy; 3 Unit of Infectious and Tropical Diseases, Department of Clinical and Experimental Sciences, University of Brescia, Brescia, Italy; 4 Institute of Microbiology, Department of Molecular and Translational Medicine, University of Brescia, ASST Spedali Civili, Brescia, Italy

**Keywords:** Risk factors, Haematological diseases

## To the Editor:

Cytomegalovirus (CMV) reactivation is observed in 40–70% of the patients following allogeneic stem cell transplantation (allo-SCT) and, together with donor CMV positive serostatus, it has been associated with impaired outcome in terms of overall survival (OS) [1, 2], and also graft versus host disease (GVHD) in several studies [3, 4].

Letermovir (LET) is the first anti-viral drug licensed for CMV prophylaxis, that has proved to be very effective in reducing the incidence of CMV clinically significant infections (CMV-csi) and CMV disease, together with an excellent safety profile. The results of the registrative study [5] have been confirmed in several single and multicentric experiences [6–9] and a recent metanalysis showed that LET significantly reduces both CMV-related events, but also mortality for all causes and non relapse mortality (NRM) [9]. On the other hand, the potential role of LET prophylaxis in reducing acute and/or chronic GVHD is still a matter of debate.

In this retrospective study, we analyzed 480 transplants consecutively performed in our Institution (NO LET ERA: *n* = 325 - March 2006/November 2018 and LET ERA: *n* = 155 - December 2018/September 2022). The management of CMV at our center was conducted as previously published [9]. Moving from the NO LET ERA to the LET ERA, we observed an increase in the transplants for primary myelofibrosis (4% vs 19%; *p* = 0.002), in haploidentical donors (11% vs 30%; *p* = 0.007) which was counterbalanced by a reduction in sibling donors (40% vs 19%; *p* = 0.008), an increase in the use of peripheral blood stem cells (PBSC) (74% vs 90%; *p* = 0.0002), and an increase in the use of myeloablative conditioning regimens (MAC) (43% vs 63%; *p* = 0.04). Moreover, we registered a significant increase in patients with HCT-CI ≥ 3 (34% vs 48%; *p* = 0.007) and a corresponding reduction in patients with HCT-CI = 0 (36% vs 24%; *p* = 0.01).

**Fig. 1:**
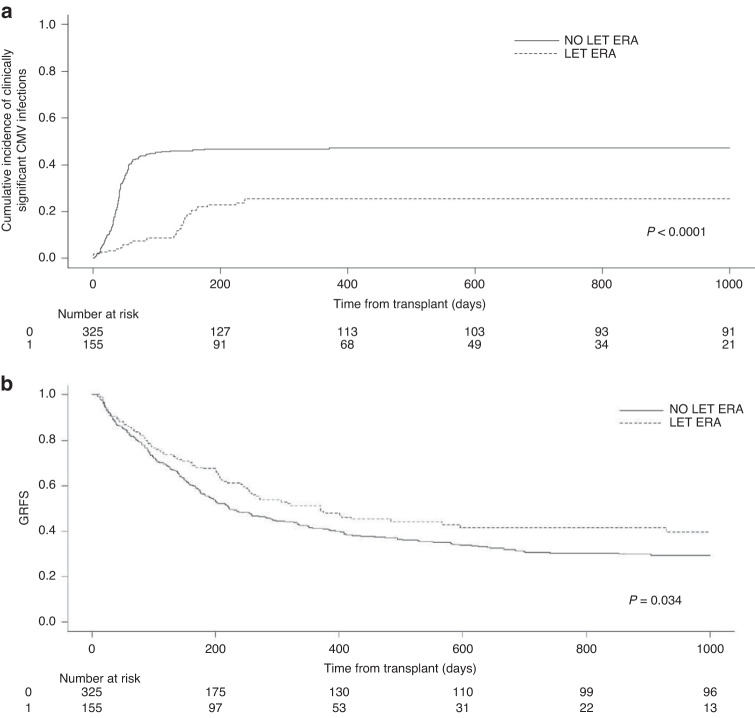
Cumulative incidence of clinically significant CMV infections (CMV-csi) and graft and relapse-free survival (GRFS) in the NO LET ERA vs LET ERA. **a** Cumulative incidence of CMVcsi at 100 days: 43% vs 8%; at 180 days: 47% vs 26%. **b** GRFS at 1 year: 49% vs. 41%.

As expected, the cumulative incidence of CMV-csi was lower in the LET ERA vs the NO LET ERA, both at day + 100 (8% vs 43%; *p* < 0.001) and at day + 180 (26% vs 47%; *p* < 0.001) (Fig. 1a). Similarly, the incidence of CMV disease declined from 6 and 8% to 1 and 3% in the two ERAs, by day + 100 (*p* = 0.02) and by day +180 (*p* = 0.02), respectively. Interestingly, in the LET ERA we registered a significant reduction in the incidence of grade II/IV aGVHD (37% vs 28%; *p* = 0.005), overall cGVHD (30% vs 12%; *p* = 0.04) and moderate/severe cGVHD (21% vs 10%; *p* = 0.003). Comparing the NO LET vs LET ERA, the 1-year NRM, the 1-year cumulative incidence of relapse (CIR) and the 1-year overall survival (OS) were 19% vs 15%; (*p* = 0.28), 31% vs 23% (*p* = 0.12), and 65% vs 73% (*p* = 0.47), respectively. The 1-, 2-, and 3-year graft and relapse free survival (GRFS) in the LET vs NO LET ERA were 41% vs 49%, 29% vs 39%, and 26% vs 39%, respectively (*p* = 0,0034; Fig. 1b). By multivariate analysis, and focusing on aGVHD the factors associated with reduced risk were: RIC regimen (HR 0.71; *p* = 0.01), sibling donor (HR 0.70; *p* = 0.02), and LET administration (HR 0.68; *p* = 0.02). In the case of cGVHD, we observed that sibling donor (HR 1.60; *p* = 0.01) was associated with increased risk, whereas LET administration was protective (HR 0.54; *p* = 0.02). When we looked at the NRM, two factors were significantly associated with reduced risk of death for causes other than relapse: low HCT-CI (HR 0.52; *p* = 0.01) and KPS 90–100 (HR 0.46; *p* = 0.02). As concerns relapse risk, the only factor that proved to protect against relapse was having received LET prophylaxis (HR 0.67; *p* = 0.05). Finally, focusing on OS, age as a continuous variable was associated with impaired outcome (HR 1.02; *p* = 0.002), whereas low-risk HCT-CI category (HR 0.66; *p* = 0.005) and a KPS 90–100 (HR 0.54; *p* = 0.01) were associated with improved long-term survival.

In this manuscript, we confirm that LET significantly reduces the incidence of CMV-csi (*p* < 0.0001; Fig. 1a). We also observed a significant reduction in the incidence of grade II/IV aGVHD, of overall cGVHD and of moderate/severe cGVHD. Thus, the use of LET was associated with a significant improvement in the 1-, 2- and 3-years graft and relapse free survival (GRFS) (*p* = 0.0034; Fig. 1b). This is of note, because the 155 patients in the LET ERA were at higher risk of developing GVHD and also CMV-related complications as compared to the patients in the NO LET ERA, because of the greater number of haploidentical transplants and greater use of PBSC [10]. If it is true that LET was independently associated with a reduced risk of cGVHD by multivariate analysis, it should be considered that two other factors may have contributed to the reduction of cGVHD in the LET ERA: the extensive use of ATG in sibling transplants with PBSC after 2016 and of post-transplant cyclophosphamide (PTCY) from 2018 onwards for haploidentical transplants. In particular, the routinely use of ATG in our center for sibling transplants with PBSC was implemented only after the results of the study by Kroger and Colleagues, in which we also enrolled patients [10]. This means that, before 2016, patients transplanted from a sibling donor did not receive any lymphodepletion and this may explain the paradoxic result of the multivariate analysis, showing that sibling donor was an independent risk factor for cGVHD.

In order to identify some indirect effect of LET use, we also compared the incidence of fever of undetermined origin (FUO), bacterial infections, proven/probable invasive fungal infections (IFIs) and re-hospitalization in the two ERAs. Although we observed a reduction in these events following the use of LET, these differences did not reach the statistical significance as in other reports [11]. Notably, in the more recent LET ERA, patients were at higher risk of developing infections (older age, more primary myelofibrosis, higher HCT-CI, more haploidentical donors, fewer sibling donors, more cases of PBSC and MAC regimens). From this viewpoint, the slight reduction in infections and re-hospitalization in the LET ERA is clinically highly significant, in particular considering the higher prevalence of high HCT-CI. We are conscious of the fact that these data are the results of a retrospective analysis, covering a long period of time, but these patients are all consecutive, and this gives a clear picture of the impact of LET prophylaxis in real-life.

Focusing on the costs of an extensive prophylaxis with LET, there are recently published data suggesting that LET is cost-effective compared with PET alone in terms of quality-adjusted life-years [12]. We calculated the costs of 1st year hospital-readmissions in the two ERAs due to transplant-related complications (namely GVHD and/or infections). In our series we did not observe any difference comparing the LET ERA and NO LET ERA. The main reason for this finding is probably represented by the fact that patients in the LET ERA were at higher risk of transplant-related complications, due to higher age and comorbidity index. This may have increased their management costs, and, may have mitigated the advantages of LET prophylaxis in costs reduction due to CMV-csi reduction.

In conclusion, we confirmed that LET prophylaxis significantly reduces the incidence of CMV-csi (Fig. 1a). Following LET introduction, we also observed a reduction of the incidence of aGVHD, overall cGVHD and moderate/severe cGVHD, and, moreover and for the first time, a significant improvement of the composite transplant outcome of GRFS (Fig. 1b).

## Data Availability

The datasets generated during and/or analyzed during the current study are available from the corresponding author on reasonable request.
