# Correction: CMV prophylaxis with letermovir significantly improves graft and relapse free survival following allogeneic stem cell transplantation

**DOI:** 10.1038/s41409-023-02158-2

**Published:** 2023-11-29

**Authors:** Michele Malagola, Vera Radici, Mirko Farina, Simone Pellizzeri, Filippo Spoldi, Enrico Morello, Nicola Polverelli, Eugenia Accorsi Buttini, Simona Bernardi, Federica Re, Alessandro Leoni, Liana Signorini, Arnaldo Caruso, Domenico Russo

**Affiliations:** 1 Blood Diseases and Cell Therapies Unit, Bone Marrow Transplant Unit, “ASST-Spedali Civili” Hospital of Brescia; Department of Clinical and Experimental Sciences, University of Brescia, Brescia, Italy; 2 Centro di Ricerca Emato-oncologico AIL (CREA), “ASST-Spedali Civili” Hospital, Brescia, Italy; 3 Unit of Infectious and Tropical Diseases, Department of Clinical and Experimental Sciences, University of Brescia, Brescia, Italy; 4 Institute of Microbiology, Department of Molecular and Translational Medicine, University of Brescia, ASST Spedali Civili, Brescia, Italy

**Keywords:** Risk factors, Haematological diseases

Correction to: *Bone Marrow Transplantation* 10.1038/s41409-023-02124-y, published online 19 October 2023

In the online version of this article, the addresses have been assigned as follows:

Michele Malagola^1,*^, Vera Radici^1^, Mirko Farina^1^, Simone Pellizzeri^1^, Filippo Spoldi^1^, Enrico Morello^1^, Nicola Polverelli^1^, Eugenia Accorsi Buttini^1,2^, Simona Bernardi^1,3^, Federica Re^1,3^, Alessandro Leoni^4^, Liana Signorini^2^, Arnaldo Caruso^1^ and Domenico Russo^1^

^1^Blood Diseases and Cell Therapies Unit, Bone Marrow Transplant Unit, “ASST-Spedali Civili” Hospital of Brescia; Department of Clinicaland Experimental Sciences, University of Brescia, Brescia, Italy.

^2^Institute of Microbiology, Department of Molecular and Translational Medicine, University of Brescia, ASST Spedali Civili, Brescia, Italy.

^3^Centro di Ricerca Emato-oncologico AIL (CREA), “ASST-Spedali Civili” Hospital, Brescia, Italy.

^4^Unit of Infectious and Tropical Diseases, Department of Clinical and Experimental Sciences, University of Brescia, Brescia, Italy.

This has now been corrected and the correct assignment is as follows:

Michele Malagola^1^ ((Envelope)), Vera Radici^1^, Mirko Farina^1^, Simone Pellizzeri^1^, Filippo Spoldi^1^, Enrico Morello^1^, Nicola Polverelli^1^, Eugenia Accorsi Buttini^1^, Simona Bernardi^1,2^, Federica Re^1,2^, Alessandro Leoni^1,2^, Liana Signorini^3^, Arnaldo Caruso^4^, Domenico Russo^1^

^1^Blood Diseases and Cell Therapies Unit, Bone Marrow Transplant Unit, “ASST-Spedali Civili” Hospital of Brescia; Department of Clinical and Experimental Sciences, University of Brescia, Brescia, Italy.

^2^Centro di Ricerca Emato-oncologico AIL (CREA), “ASST Spedali Civili” Hospital, Brescia, Italy.

^3^Unit of Infectious and Tropical Diseases, Department of Clinical and Experimental Sciences, University of Brescia, Brescia, Italy.

^4^Institute of Microbiology, Department of Molecular and Translational Medicine, University of Brescia, ASST Spedali Civili, Brescia, Italy.

The original article has been corrected.

